# New drug regulations in France: what are the impacts on market access? Part 1 – Overview of new drug regulations in France

**DOI:** 10.3402/jmahp.v1i0.20891

**Published:** 2013-08-06

**Authors:** Cécile Rémuzat, Mondher Toumi, Bruno Falissard

**Affiliations:** 1 Creativ-Ceutical, Paris, France; 2 Department of Decision Sciences and Health Policies, University Claude Bernard Lyon 1, UFR d'Odontologie, Lyon, France; 3 INSERM Unit U669 (Public Health and Mental Health), University Paris-Sud, Maison de Solenn, Paris, France

**Keywords:** France, market access, drugs, law, pricing, reimbursement, comparative evidence, medico-economic assessment

## Abstract

Access to the French drug market is being impacted by an ongoing dramatic shift in practice as well as by two laws that came into force in December 2011. This new environment has been described and analyzed in two separate articles. The first article aims to describe the recent changes in access to the French drug market. The severity of the condition being treated, which used to be the main determinant of the drug's reimbursement level in France, has now been replaced with the drugs’ efficacy criterion. Moreover, the effect size required for acknowledging drug innovation has substantially increased. Perceived evidence might also be more important than actual evidence. Comparative evidence and real-world data are considered critical conditions for marketing authorization. Cost-effectiveness studies will now be part of the market access requirements for all drugs in order to satisfy the selection criteria for medico-economic assessment.

The current process of access to the French drug market is experiencing a shift in its health technology assessment (HTA) practice, although this has never been officially reported; the process will be impacted by further changes in the years to come, following two bills that were passed in 2011: the law for the reinforcement of the health safety of drug and health products (1), and the Social Security Funding Law for 2012 (2). The objective of this article is to describe these recent changes in access to the French drug market. The impact of these changes is presented in a separate article, ‘New Drug Regulations in France: What Impacts on Market Access? Part 2 – Impacts on Market Access and for the Pharmaceutical Industry’.

## Overview of market access for medicines in France

### Marketing authorization

In France, marketing authorization for drugs can be obtained at the national or European level. The French regulatory agency for drugs is the National Agency for the Safety of Medicine and Health Products (Agence Nationale de Sécurité du Médicament et des Produits de Santé, or ANSM), previously known as the French Agency for the Medical Safety of Health Products (Agence Française de Sécurité Sanitaire des Produits de Santé, or AFSSAPS). In Europe, the regulatory agency for drugs is the European Medicines Agency (EMA).

### HTA, reimbursement, and pricing

The Transparency Committee (Commission de la Transparence, or CT) is one of the scientific committees of France's HTA agency, the French National Authority for Health (Haute Autorité de Santé, or HAS), providing scientific advice concerning the usefulness, interest, and appropriate use of drugs. The CT is in charge of assessing the medical benefit (*service médical rendu*, or SMR) and the improvement of medical benefit (*amélioration du service médical rendu*, or ASMR) of a new medicine for which a pharmaceutical company submits a request for inclusion in the reimbursable drugs formulary (3, 4).

Drug price setting is established by the Economic Committee on Healthcare Products (Comité Economique des Produits de Santé, or CEPS) after negotiation with the drug company. ASMR is one of the key items taken into account during price setting.

The reimbursement rate is fixed by a decision of the National Healthcare Insurances (Union Nationale des Caisses d'Assurance Maladie, or UNCAM) based on SMR.

The Health Ministry makes the final decision regarding whether or not the drug will be registered on the list of reimbursable medicines. This registration is valid for 5 years. At the end of this period or at any time when significant new information becomes available, the CT reevaluates the SMR and ASMR levels (3, 4).

The SMR represents the medical value of a drug, taking into account the severity of the disease as well as the data regarding the drug: its clinical efficacy, effectiveness, and safety; the position of the medicine in the therapeutic strategy, and the existence or absence of therapeutic alternatives; the type of treatment (preventive, curative, or symptomatic); and the public health impact (e.g., the burden of the disease or its impact on the healthcare system). There are five SMR levels, for which UNCAM applies different reimbursement rates:

1. Major: 100% reimbursement for medicines recognized as irreplaceable and expensive ones
2. Important: 65% for medicines with important or major SMR
3. Moderate: 30% (changed from 35% in May 2011) for medicines with moderate SMR
4. Weak: 15% for some medicines with weak SMR
5. Insufficient: Drugs with insufficient SMR are not included on the list of reimbursable drugs.

See also Tables I and II (3–7).

**Table I:** Five items taken into account to assess SMR

Severity of the disease
Clinical efficacy, effectiveness, and safety
Position of the medicine in the therapeutic strategy, and the existence or absence of therapeutic alternatives
Type of treatment (preventive, curative, or symptomatic)
Public health impact

**Table II:** Five levels of SMRs and their levels of reimbursement

Level of SMR	Level of reimbursement (%)
Major	100*
Major or important	65
Moderate	30
Weak	15
Insufficient	0

* For medicines recognized as irreplaceable and especially expensive.

In France, the percentage of individuals covered by private health insurance is about 95% (8), and public health insurance expenses are shifting toward private health insurance, resulting in a continuous increase in copayments.

The ASMR is based on the degree of clinical improvement of the new medicine relative to any existing treatments (i.e., its clinical added value), usually the best next alternative. There are five ASMR levels, as described in Table III (3, 4).

**Table III:** Five levels of ASMR

Level of ASMR	Criteria
I	Major innovation: innovative product with substantial therapeutic benefit
II	Important improvement in terms of therapeutic efficacy and/or reducing side effects
III	Moderate improvement in terms of therapeutic efficacy and/or utility
IV	Minor improvement in terms of therapeutic efficacy and/or reducing side effects
V	No improvement over existing options but still can be recommended for reimbursement (e.g., generic drugs and me-too drugs)

The ASMR is mainly driven by the effect size of the benefit of the drug. Although the effect size concept is a well-defined and standardized measure of the benefit over a comparator (9), in France, the effect size is considered by the CT as a very subjective endpoint that relies on the subjective expert assessment of the CT members (P. Bouvenot, 2010, oral communication). The lack of decision analysis framework leaves some unpredictability.

## Shift in HTA practices

Over the past few years, SMR and ASMR appreciation has dramatically shifted:

1. *Regarding SMR*: Originally, the severity of the condition targeted by a drug determined the SMR score. Today, the criterion of drug efficacy is increasing in importance to the detriment of the criterion of disease severity (7).
2. *Regarding ASMR*: The effect size required to achieve a given ASMR has increased substantially in recent years (5, 10).

Also, it is worth noting that perceived evidence-based assessment, rather than absolute evidence, seems to play an important role in the decision-making processes of the CT. We can cite the example of the reassessment of the SMR and ASMR of Alzheimer's disease treatments. The appraisals of the CT were reviewed for three acetylcholinesterase inhibitors [donepezil (Aricept^®^), galantamine (Reminyl^®^), and rivastigmine (Exelon^®^)] and an NMDA (N-methyl-D-aspartate) receptor antagonist [memantine (Ebixa^®^)], from marketing authorization to October 2011. In the absence of any new clinical evidence, the SMR and ASMR had been downgraded: the SMR decreased from important to weak, and the ASMR decreased from level II to level V. These reassessments suggest a change in appreciation of the same benefit over time (11, 12).

In 2011, a reform of the SMR–ASMR system was under discussion within the HAS. The HAS had worked on a new relative therapeutic index to merge the SMR and ASMR. According to the chairman of HAS, two previous indexes entitled ‘SMR’ and ‘ASMR’ made the situation too complex to understand and multiplied the combinations; moreover, ANSM, the new mission of the French drug agency, by assessing the benefit–risk ratio through the life cycle of the product had led to the disappearance of the SMR notion because a medicine with no medical benefit could not obtain a marketing authorization. A four-level multiattribute index that guided reimbursement level and price setting was initially suggested (categorizing drugs into insufficient, moderate, important, and major levels), based on its benefit over the available products, the severity of the disease, alternative treatments, the interest of a patient subgroup, pharmaceutical formulation, and other attributes (13). This new index, called the Relative Therapeutic Index (Index Thérapeutique Relatif, or ITR), was then described in the HAS annual report of 2011 as a five-level index driving pricing and reimbursement based on the analysis of comparative data (14). In this index, a drug should be scored between −1 and 3 or more, depending on its relative efficacy assessment eligibility, its relevant competing product(s), the study endpoints, and the study methodology, and modulated by its safety and the conditions of use (Table IV) (HAS, oral communication, January 2013). This reform was expected to come into force initially in 2012 via internal regulation revision (not law), as indicated by the chairman of HAS chairman during a communication reported in December 2011 (13). Then, this reform was expected to be integrated in the Social Security Funding Law (Loi de Financement de la Sécurité Sociale, or LFSS) for 2013 (14), but eventually was not included (15). This new index is still being discussed within the HAS and could be implemented in 2014.

**Table IV:** Relative Therapeutic Index (ITR) driving pricing and reimbursement

ITR		Level of reimbursement	Price
−1	Inferiority compared to the relevant comparator	None	–
	Non-relevant comparator		
	Unacceptable methodology		
	Lack of proof		
0	Non-inferiority compared to the relevant comparator	Same as that of the comparator	Article R 163-5 (a price that will enable expenditure savings versus the comparator)
1	Minor improvement compared to the relevant comparator	Same as that of the comparator	Framework agreement with CEPS
	Improvement of conditions of use with impact on care and non-inferiority compared to the comparator		
2	Moderate improvement compared to the relevant comparator	Same as that of the comparator	Framework agreement with CEPS
≥3	Major improvement compared to the relevant comparator	Same as or more than that of the comparator	Framework agreement with CEPS

Moreover, based on its actual decisions, the CT edited for the first time an ex-post analysis decision framework in the HAS annual report of 2011. This framework is based on the review and analysis of a previous decision. Notably, the CT has worked on criteria to obtain insufficient SMRs and identified several cases for non-reimbursement, as summarized in Table V (14).

**Table V:** Seven reasons for non-reimbursement identified by the CT

Very low-relevance or non-relevant efficacy, with substantial side effects, despite a favorable benefit–risk ratio
Lack of evidence of efficacy
Efficacy evidenced only for off-label use or in a population where efficacy could not be transferred to the actual population
Lack of room in the therapeutic strategy
Targets benign symptom, disease, or spontaneous recovery
Less effective and/or less safe than available treatment
Justification of fixed combinations not proved

## New drug safety law (no. 2011–2012 of 29 December 2011) related to reinforcing the health safety of drugs and health products

The law related to reinforcing the health safety of medicine and health products aims to reorganize the safety monitoring of health products in order to reconcile patient safety with access to therapeutic innovation (1). The main changes in this law impact the Public Health Code and Social Security Code. However, a substantial number of articles just transpose a recent (2010) European pharmacovigilance directive (16).

The bill was provided for a first reading to the National Assembly of France on August 1, 2011, and subjected to an accelerated procedure begun by the government on September 19, 2011. This law was definitively adopted on December 19, 2011, and published in the French gazette on December 30, 2011, (17). To date, application decrees have only been partially published (18).

### Context of the law

The need for a law that would reinforce the safety of drugs and health products emerged from the late withdrawal from the market of benfluorex, a drug accused of leading to the deaths of 500–2,000 patients (19). Benfluorex had been marketed since 1976 and was withdrawn from the market only on November 30 2009, despite its known risks of inducing cardiac valvulopathy and pulmonary arterial hypertension. In November 2010, the Minister of Health requested the General Inspection of Social Affairs (Inspection Générale des Affaires Sociales, or IGAS) to investigate the case of benfluorex to better understand the sequence of events that occurred, the decision-making process from the drug's marketing authorization to its market withdrawal, as well as the roles and responsibilities of the organizations and persons involved in this process.

The results of the investigation by IGAS (20) pointed out not only the responsibilities of the marketing authorization holder but also the failures in functioning of the French healthcare system, notably the unexplained tolerance of the French drug agency for a medicine without a real therapeutic efficacy and the inability of the pharmacovigilance system to analyze the serious risks that appeared in terms of the drug's cardiotoxicity. This report claimed that AFSSAPS had been overburdened with work, rendered inefficient by bureaucratic procedures, and constrained by fear of legal disputes with companies. AFSSAPS's failures were also attributed to its conflicts of interest and to industry influence over the agency.

The CT, on two occasions (November 17, 1999, and May 10, 2006), stated that benfluorex had an insufficient SMR and did not favor reimbursement of this drug. However, this drug was also not registered on the list of medicines suggested for de-reimbursement, as the CT was waiting for the conclusions of a reassessment of the benefit–risk ratio of AFSSAPS (21).

At the request of AFSSAPS, a prospective study named REFLEX is ongoing to assess, by ultrasound, the progress of the valvulopathy of a cohort of about 1,000 patients treated by benfluorex between 2006 and 2009 (22).

The various investigations performed following this event evidenced the need to restore confidence in the French health safety system regarding health products, particularly in the field of drugs.

These works highlighted the main issues to be addressed in the bill:

1. Guarantee better transparency regarding conflicts of interest;
2. Optimize the governance of the agencies involved in the drug sector;
3. Adapt the procedure and conditions of marketing authorization;
4. Guide the prescribers in a better way in their therapeutic choices;
5. Reinforce the drug-monitoring system;
6. Disseminate good-quality information on the health products to the public and health professionals;
7. Reinforce the supervision of medical devices.

Moreover, this bill was set:

1. in a French context of:
  - Overconsumption of medicines negatively affecting public health and the health insurance system;
  - The importance of promotional activities by the pharmaceutical industry;
  - The importance of off-label prescriptions;
  - Underreporting adverse events related to health products.
2. to fit with European and international requirements in the drug sector, especially to transpose ‘Directive 2010/84/EU (16) of the European Parliament and of the Council of December 15, 2010, amending, as regards pharmacovigilance, Directive 2001/83/EC on the Community code relating to medicinal products for human use’; and ‘Directive 2011/62/EU (23) of the European Parliament and of the Council of June 8, 2011, amending Directive 2001/83/EC on the Community code relating to medicinal products for human use, as regards the prevention of the entry into the legal supply chain of falsified medicinal products’ (24).

## Overview of dispositions of the law impacting market access

### Reinforcement of transparency regarding conflicts of interest

The transparency of conflicts of interest constitutes the first title of this law.

A declaration of conflicts of interest (direct or indirect) has to be established by health experts; the members of committees and councils attached to the ministers in charge of health and social security; the members of the Cabinet of Ministers; the managers, managerial and supervisory staff; and the members of collegiate bodies, committees, work groups, and councils of authorities and organizations, including ANSM, HAS, and the Regional Health Agency (Agence Régionale de Santé, or ARS) (Article 1).

Moreover, the companies that manufacture or market health products and the companies ensuring the services associated with these products are obliged to publish the benefits that their product or service provides; these companies have to make public their agreements established with any persons, associations, establishments, foundations, societies, and organizations involved in the health or telecommunication sector as well as direct and indirect cash and non-cash benefits beyond a certain threshold. This part of the law was called the French Sunshine Act, in reference to the American initiative of March 2010 entitled the Physician Payments Sunshine Act that requires the manufacturers of drug, device, biologics, and medical supplies to report any payments (cash or non-cash, such as gifts) made to physicians or teaching hospitals (24) (Article 2). Criminal penalties are imposed in cases of non-compliance with these transparency requirements (Article 4).

### Substitution of AFSSAPS by ANSM and reinforcement of the pharmacovigilance system with the transposition of European Directive 2010/84/EU of December 15, 2010, related to pharmacovigilance of drugs

AFSSAPS became ANSM, and its administrative board and responsibilities were redefined. Moreover, ANSM acquired a general health-policing power, which included the ability to impose financial penalties (Article 5).

The redefined administrative board included new members: three members of parliament and three senators, some representatives of compulsory basic health insurance regimens, some representatives of health professionals who have a right to prescribe and dispense medicines and health products, and some representatives of approved associations (Article 7).

ANSM took over the previous activities of AFSSAPS and had extended responsibilities: the assessment and reassessment of benefits and risks related to the use of health and cosmetic products, implementation of the pharmacovigilance system, and right to compulsorily request comparative clinical trials (Article 5).

Regarding pharmacovigilance reinforcement, this law transposes Directive 2010/84/EU of the European Parliament (16) and of the council of December 15, 2010, amending, as regards pharmacovigilance, Directive 2001/83/EC on the community code relating to medicinal products for human use (Article 28).

### Reinforcement of follow-up of medicines after marketing authorization

The reinforcement of follow-up of medicines after they receive marketing authorization is addressed through several dispositions of this law:

1. Possibilities for ANSM to require additional studies after marketing authorization: these can include safety and efficacy postauthorization studies (in comparison with available reference treatments when they exist) and also a specific follow-up of the medicine's ‘potential’ risks, complications, and medico-social management, via a patient registry, when the medicine, even if withdrawn, is susceptible to leading to a serious adverse event (Article 9).
2. Specification of the criteria leading to suspension, withdrawal, or changes in the medicine's marketing authorization, or to a dispensation and prescription prohibition: this occurs if the medicine is harmful, does not have therapeutic results, or has an unfavorable benefit–risk ratio; if the qualitative and quantitative composition has not been declared; if the requirements related to manufacturing authorization were not respected; or if the conditions of marketing authorization are not respected, especially those concerning postauthorization studies and pharmacovigilance. These decisions are made public (Article 11).
3. Establishment of communication rules for assessment of the benefit–risk ratio of the medicines:
  - The marketing authorization holder is obliged to inform ANSM of any prohibition or restriction imposed by other competent authorities regarding its medicines or health products, and of any new information that could affect the evaluation of benefits and risks of the medicine or health product.
  - ANSM can require, at any time from the marketing authorization holder, the transmission of data showing that the benefit–risk ratio remains favorable.
    Any marketing authorization holder that stops marketing its drug in another country than France has to inform ANSM and provide the reason (Article 12).
4. Monitoring of the real use of drugs: pharmaceutical companies have to ensure the proper use of the medicines in its portfolio, take appropriate measures in cases of non-conformity regarding prescriptions, and inform ANSM. ANSM can impose financial penalties on pharmaceutical companies if they do not report an adverse event, do not transmit new information that could affect the evaluation of benefits and risks of the medicine or health product, or do not take appropriate measures in cases of detection of bad use of medicines (Article 31).
5. Introduction of the notion of whistleblowers to report adverse events, and their protection in terms of discrimination (Article 43).

### Comparative evidence for reimbursement

Article 14 of the law specifies that the registration of a medicine on the list of reimbursable drugs depends on the setup of comparative clinical trials versus reference therapeutic strategies (when these exist).

### Implementation of a framework for off-label prescribing

The off-label prescription of a medicine is authorized in some circumstances:

1. if there is no appropriate therapeutic alternative;
2. if the considered indications or the conditions of use were the subject of a temporary recommendation for use (*recommandation temporaire d'utilisation*, or RTU), established by the ANSM; or
3. if the prescriber considered the medicine as essential, regarding the current scientific data, to improve or stabilize the clinical state of the patient.

The prescriber has to inform the patient that he or she is receiving an off-label prescription of the medicine, of the benefit–risk ratio, and of the coverage level by health insurance. The prescriber has to motivate his or her prescription in the medical file and to report explicitly ‘off-label prescription’ on the prescription form designated for the pharmacy delivery (Article 18).

The reimbursement of products that are prescribed off-label is possible only for a limited period, that is, if they are included in an opinion or a recommendation of the HAS after the consultation of ANSM (Article 27).

### Modification of the framework of authorization for temporary use (autorisation temporaire d'utilisation, or *ATU*)

In France, a legal framework (the ATU) addresses the use of non-approved drugs. In order to prioritize the authorization of cohort ATUs, prioritize the implementation of clinical trials in France, and reinforce the monitoring of patients treated by medicines having a named-patient ATU (i.e., an ATU nominative), named-patient ATU authorizations are restricted (Article 26).

### Reinforcement of the development and use of generic drugs

Article 42 of the law specifies that in cases of patents on the appearance and texture of oral presentations of a reference drug, the oral presentation of a generic drug with the same or similar appearance and texture cannot be forbidden.

Article 32 of the law specifies that the name of active compounds of the drug must be reported on the prescription form to improve patient information [an international non-proprietary name (INN) first, a name reported in the French or European pharmacopoeia, or a common usual name]. It can also be associated with the brand name of the drug. Until now, the INN prescription was mandatory for medicines registered in the generic group repertory (Article L5125-23 of France's Public Health Code).

## Social Security Funding Law for 2012: Law no. 2011–1906 (21 December 2011)

This bill was provided for a first reading to France's National Assembly on October 15, 2011 (2). It was definitively adopted as law on November 29, 2011, and published in the French gazette on December 22, 2011 (25).

### Context of the law

In 1996, France's LFSS was implemented to control the social and health budget. It determines the conditions needed to maintain financial balance of the Social Security system and determines the budget objectives based on a forecast of revenues. This law is voted on each year (26).

Pressures to confront the Social Security system's mounting healthcare account deficit have augmented as a result of the current economic downturn. This has prompted the French government to adopt austerity measures impacting the drug market.

The LFSS for 2011, which was adopted in November 2010, included a range of measures, including cutting prices for innovative and generic products, encouraging generic drug use, and issuing reimbursement rate cuts that were expected to generate €500 million of savings in 2011. This had a negative impact on the size of the French pharmaceutical market (27).

The LFSS for 2012 pursued its efforts in consolidating social accounts, which started with the LFSS for 2011.

### Overview of the dispositions adopted to control health insurance expenses

The global deficit of the Social Security system (all regimens) was set at €20.1 billion in 2011, and the objective for 2012 was set at €15.6 billion. The French government has reinforced its efforts in terms of health insurance to reduce the deficit set at €9.5 billion in 2011 to €5.8 billion in 2012.

The initial contribution of the pharmaceutical industry in the reduction of expenses had been set at €770 million. On November 7, 2011, the French government presented the measures of the public finances plan that will have a greater impact on this contribution in terms of drug expenses, to reach additional savings of €290 million.

The main economic dispositions to control health insurance expenses that were adopted for 2012 in the pharmaceutical industry included the following measures:

1. Decreasing the price of health products (negotiated through conventions between CEPS and the pharmaceutical industry)
2. Setting a reference price for branded products that have lost protection but have not reached substantial generic penetration (*tarif forfaitaire de responsabilité*, or TFR)
3. Decreasing wholesalers’ margins
4. De-reimbursing medicine with insufficient SMRs
5. Maintaining the K rate at 0.5%. Rebates due by the pharmaceutical companies subject to the safeguard clause, based on their turnovers, are determined by K rates. If a K rate is exceeded, payback is due by the pharmaceutical companies.
6. Increasing the tax on the turnover of the pharmaceutical industry to 1.6% for the period 2012–2014 (versus 1% for the period 2009–2011)
7. Extending the tax on promotional expenses, including costs on advertising in the medical press
8. Increasing the taxes on marketed medicines and health products, mainly to finance the work of ANSM
9. Reinforcing the medico-economic assessment of the health products

Since the LFSS for 2008, HAS must produce ‘medico-economic opinions’ to determine the most efficient therapeutic strategies and edit recommendations accordingly. The Economic and Public Health Assessment Committee (Commission Evaluation Economique et de Santé Publique, or CEESP) was set up to comply with this mission; however, until now the works and opinions from this committee did not impact the pricing and reimbursement of health products, as initially planned. Moreover, this committee did not exist as a legal entity and was not described in the Social Security Code, but just as an internal working group to HAS. However, the LFSS for 2012 includes CEESP in the Social Security Code as a specialized committee in charge of providing recommendations and medico-economic opinions in Article 47 (25, 28–30). The current structure of CEESP has been adapted to comply with this mission (14).

**Fig. 1:**
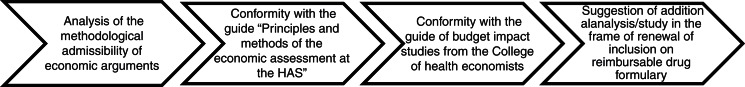
Flash opinion of CEESP.

Decree no. 2012-1116 of October 2, 2012 (31), related to the medico-economic missions of the HAS, specified the cases in which a medico-economic assessment will be required for drugs. It will be applicable from October 2013. Two criteria must be met: 1) The ASMR of the drug claimed by the company is major, important, or moderate (ASMR I, II, or III, respectively); and 2) the drug is likely to have a significant impact on the health insurance budget regarding its impact on care organization, professional practices, or patient care and, when applicable, its price. This decree also specifies the medico-economic assessment procedure. The pharmaceutical company, together with its request for inclusion (or renewal of inclusion) of a medicine on the reimbursable drugs formulary, transmits all medico-economic data related to the drug to CEESP and CEPS. CEESP will provide an opinion [reported as a ‘flash opinion’ (14)] (Fig. 1) on the predictable or established efficiency of the drug and its coverage by health insurance. This opinion is based on comparative analysis, between the different therapeutic alternatives, of the ratio of the cost compared to the expected or observed benefit for patient health and quality of life. The pharmaceutical company has 8 days to address its comments following the receipt of this opinion. The final opinion is sent to the pharmaceutical company and CEPS. It is also made public.

## The LFSS for 2013: Law no. 2012–1404 (December 17, December 2012)

The LFSS for 2013 pursues cost-containment measures to control health insurance expenses, with planned price cuts on brand and generic drugs, as well as actions to enhance generic uptake (32). One interesting point of this law in terms of drug market access is the possibility to adopt an RTU for an off-label drug, even if an appropriate therapeutic alternative exists, in cases of public health necessity or to avoid expenses that will have a significant impact on health insurance (Article 57).

This measure was taken by the government to counteract the commercial strategies of some pharmaceutical companies to optimize the pricing of their drugs, a practice that is responsible for high expenditures in health insurance and off-label use. Indeed, some companies have the same drug under different brand names and do not request marketing authorization for each brand for all indications; this allows the companies to have different prices for the same molecule in different indications, and only one brand on the market in one indication at a high price (33).

## Conclusion

In light of these changes, it clearly appears that the access to the French drug market will be increasingly driven by data pertaining to comparative-effectiveness and cost-effectiveness, and an increased role of postmarketing studies in the years to come. Pharmaceutical companies will need to anticipate these requirements during drug development, which will be increasingly challenging.
